# Early detection of influenza outbreak using time derivative of incidence

**DOI:** 10.1140/epjds/s13688-020-00246-7

**Published:** 2020-09-11

**Authors:** Woo-Sik Son, Ji-Eun Park, Okyu Kwon

**Affiliations:** 1 National Institute for Mathematical Sciences, 34047 Daejeon, South Korea; 2 Korea Institute of Oriental Medicine, 30675 Daejeon, South Korea

**Keywords:** Characteristics of influenza spread, Early outbreak detection

## Abstract

For mitigation strategies of an influenza outbreak, it can be helpful to understand the characteristics of regional and age-group-specific spread. In South Korea, however, there has been no official statistic related to it. In this study, we extract the time series of influenza incidence from National Health Insurance Service claims database, which consists of all medical and prescription drug-claim records for all South Korean population. The extracted time series contains the number of new patients by region (250 city-county-districts) and age-group (0–4, 5–19, 20–64, 65+) within a week. The number of cases of influenza (2009–2017) is 12,282,356. For computing an onset of influenza outbreak by region and age-group, we propose a novel method for early outbreak detection, in which the onset of outbreak is detected as a sudden change in the time derivative of incidence. The advantage of it over the cumulative sum and the exponentially weighted moving average control charts, which have been widely used for the early outbreak detection of infectious diseases, is that information on the previous non-epidemic periods are not necessary. Then, we show that the metro area and 5–19 age-group are earlier than the rural area and other age-groups for the start of the influenza outbreak. Also, the metro area and 5–19 age-group peak earlier than the rural area and other age-groups. These results would be helpful to design a surveillance system for timely early warning of an influenza outbreak in South Korea.

## Introduction

For preparedness for an influenza outbreak, we have to know what intervention strategies are effective. Therefore, there have been increasing interests for mitigation measures for influenza. In the works of [1, 2], large scale stochastic simulation models were used for investigating various control strategies: antiviral, vaccine and nonpharmaceutical (case isolation, household quarantine, school or workplace closure, restrictions on travel) measures. The authors of [3] studied the effectiveness of preventive measures for pandemic influenza in Italy by using a global compartmental model and an agent-based model. Besides reducing the infected cases and delaying the peak time, the economic impact of influenza mitigation strategies was evaluated by a stochastic agent-based model [4]. Also, the authors of [5] studied to understand how behavioral changes of individuals to intervention strategies affect the spread of infectious disease.

On the other hand, it can be helpful to understand the characteristics of regional and age-group-specific spread for control policies to an influenza outbreak. There have been several works on investigating the spatiotemporal spreading pattern of influenza in a country or regions containing a number of countries. For Japan, using Kriging analysis on influenza-like illness (ILI) data, the authors of [6] showed that the starting areas of peak ILI activity were mostly found in western Japan. Also, the wavelet analysis for sentinel surveillance data was considered for studying the spatiotemporal pattern of influenza in Japan [7]. For the U.S., using real-time syndromic surveillance systems of Massachusetts, the authors of [8] identified target age groups within the pediatric population that develop influenza the earliest and are most strongly linked with mortality in the population. In the work of [9], harmonic regression models for hospitalization records of influenza in the U.S. demonstrated that western states peaked earlier and New England states peaked later. For Europe [10] and the Middle East, North Africa regions [11], the FluNet database was used for investigating the spatiotemporal spreading pattern, respectively.

In South Korea, however, there has been no official statistic related to the characteristics of regional and age-group-specific spread of influenza. Korean Influenza Surveillance System (KISS) has reported the number of ILI cases per 1000 outpatients from 200 sentinel clinics. It has not been divided by region and age-group but has been aggregated together. Therefore, we extract the time series of influenza incidence from National Health Insurance Service (NHIS) claims database, which consists of all medical and prescription drug-claim records for all South Korean population. The extracted time series contains the number of new patients by region (250 city-county-districts) and age-group (0–4, 5–19, 20–64, 65+) within a week. Then, the number of cases of influenza (2009–2017) is 12,282,356. Note that all of the above works [6–11] on a spatiotemporal spreading pattern of influenza used sentinel surveillance data rather than the whole incidence data. Also, the above works only considered the propagation of epidemic peak and the onset of outbreak was rarely discussed. In this study, we use the whole incidence data for all South Korean population and investigate the propagation of the onset of influenza outbreak as well as the peak. For computing the start of an influenza outbreak by region and age-group, we propose a novel method for early outbreak detection called time derivative (TD) method. In the TD, the onset of outbreak is detected as a sudden change in the time derivative of incidence. The advantage of the TD over the cumulative sum (CUSUM) [12] and the exponentially weighted moving average (EWMA) [13] control charts, which have been widely used for the early outbreak detection of infectious diseases, is that information on the previous non-epidemic periods are not necessary. Then, we show that the metro area and 5–19 age-group are earlier than the rural area and other age-groups for the start of the influenza outbreak. Also, the metro area and 5–19 age-group peak earlier than the rural area and other age-groups. These results would be helpful to design a surveillance system for timely early warning of an influenza outbreak in South Korea.

The rest of this paper is organized as follows. Section 2 describes how we extract the time series of influenza incidence from the NHIS claims database by using an episode of care. In Sect. 3, we discuss the TD and show that it is more accurate than the CUSUM and the EWMA for early outbreak detection of influenza. Then, in Sect. 4, we show the results on the characteristics of regional and age-group-specific spread of influenza in South Korea. We conclude this paper in Sect. 5.

## Data

**Figure 1:**
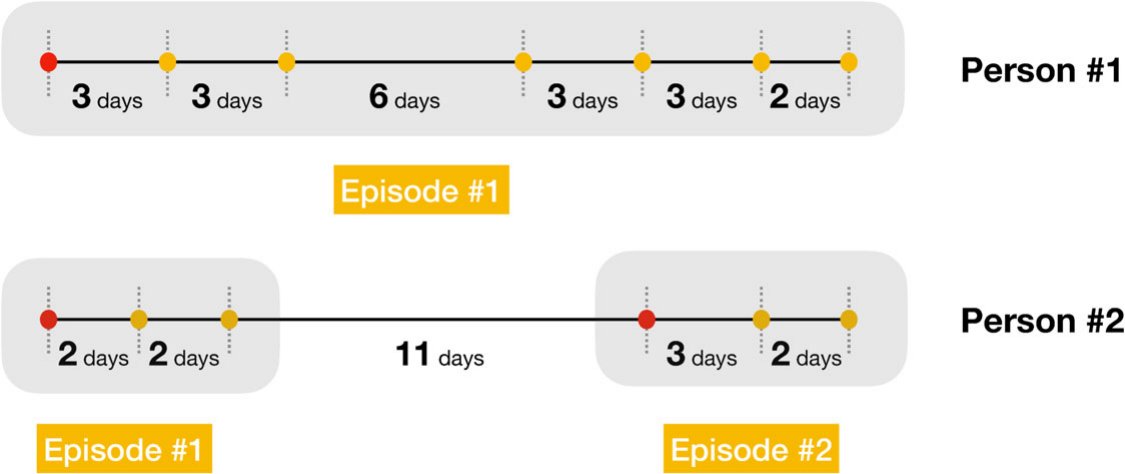
An episode of care. The medical records for influenza within 10 days from the most recent medical record of the same person are bound to a single episode of care. Then, we choose the first medical record of each episode of care, which is marked by a red dot, as an incidence of influenza. Here, yellow dots mean medical records within the same episode of care

We extract the time series of influenza incidence from the NHIS claims database, which consists of all medical and prescription drug-claim records for all South Korean population [14]. The NHIS claims database contains four data tables: general information of specification (20T), consultation statements (30T), diagnosis statements classified by the International Classification of Diseases 10th revision (ICD-10; 40T) [15], and detailed statements about prescriptions (60T) [16]. Using these data tables, the incidence time series is extracted through the following two steps. First, we collect all claim records whose diagnosis statements include ICD-10 codes for influenza, that is, J09, J10, and J11, or whose prescriptions contain influenza-specific drugs, that is, Oseltamivir and Zanamivir. Second, we generate the episode of care out of the claim records collected through the first step. The episode of care is defined as the set of services provided by a health care facility for a specific medical problem or condition or specific illness [17]. As shown in Fig. 1, the medical records for influenza within 10 days from the most recent one of the same person are bound to a single episode of care. Note that the 10 days is not the time interval between the first and last medical records of the episode of care. Then, we choose the first medical record of each episode of care, which is marked by a red dot in Fig. 1, as an incidence of influenza. Here, yellow dots mean medical records within the same episode of care. We confirm that the 10 days is sufficient to constitute the episode of care for influenza. Due to the Act on the Protection of Personal Information Maintained by Public Agencies of South Korea, when we export the incidence time series from the NHIS database, the time resolution is limited by a week, not a day. As a result, we obtain the time series of influenza incidence, which contains the number of new patients by region (250 city-county-districts) and age-group (0–4, 5–19, 20–64, 65+) within a week. Then, the number of cases of influenza (2009–2017) is 12,282,356.

## Methods

In this study, we investigate the characteristics of regional and age-group-specific spread of influenza in South Korea. Such as, in which region and in which age-group does an influenza outbreak start and peak earlier? Finding out when the peak has occurred is straightforward from the incidence time series. The peak week is retrospectively obtained from the incidence time series. That is, the maximum of time series for each season is the peak. Therefore, this section will address how to compute the start of an influenza outbreak by region and age-group. In South Korea, there have been currently three national influenza surveillance systems [18, 19]. First, the KISS explained in Sect. 1 reports the number of ILI cases per 1000 outpatients from 200 sentinel clinics. Second, Korean Influenza and Respiratory Viruses Surveillance System (KINRESS) assembles respiratory specimens from 52 sites (as of July 2018) among the clinics participating in the KISS. The KINRESS reports weekly positivity of influenza tests along with other respiratory viruses including respiratory syncytial virus, parainfluenza virus, adenovirus, human rhinovirus, human metapneumovirus, human coronavirus, and human bocavirus. Third, Hospital-based Influenza Morbidity and Mortality (HIMM) aims to monitor not only influenza activity but also influenza severity, such as hospitalization, complication, and mortality. Then, Korea Centers for Disease Control and Prevention (KCDC) issues a warning for the onset of influenza outbreak when the number of ILI cases is larger than the baseline, which is defined as the mean number of ILI cases in non-epidemic periods of three previous influenza seasons plus two standard deviations [20]. Since the number of ILI cases is not divided by region and age-group but is aggregated together, it is not possible to apply the baseline of the KCDC for computing the onset of influenza outbreak by region and age-group. The CUSUM [12] and the EWMA [13] control charts have also been widely used for the early outbreak detection of infectious diseases. They require the mean and standard deviation of incidence in the previous non-epidemic periods as well, and could not be applied for computing the onset of influenza outbreak by region and age-group in South Korea.

**Figure 2:**
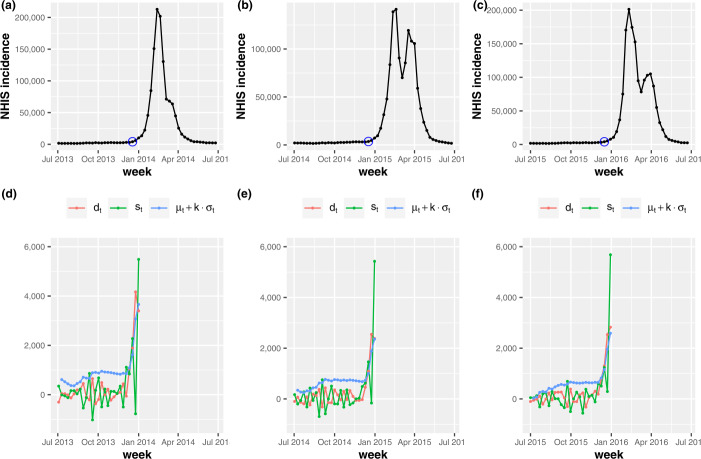
The time derivative of influenza incidence. Fig. 2(**a**)–(**c**) show the total number of new patients within a week for three influenza seasons (2013–14, 2014–15, 2015–16). In Fig. 2(**d**)–(**f**), $d_{t}$, $s_{t}$, $\mu _{t}$, and $\sigma _{t}$ represent the first derivative, second derivative, seasonal cumulative mean and standard deviation, respectively. We confirm that $d_{t}$ abruptly fluctuates around zero before the onset of influenza outbreak. Then, the difference between $d_{t}$ and $\mu _{t}$ increases at the onset of influenza outbreak. In Fig. 2(**a**)–(**c**), the onset week *t* where the condition (2) is satisfied is marked by the blue circle

Then, we propose a novel method for early outbreak detection, i.e., the TD, which does not require information on non-epidemic periods of the previous seasons. In the TD, the start of an influenza outbreak is detected as a sudden change in the time derivative of incidence. Figure 2(a)–(c) show the total number of new patients $y_{t}$ in South Korea within a week *t* for three influenza seasons (2013–14, 2014–15, 2015–16). In Fig. 2(d)–(f), $d_{t}$ and $s_{t}$ represent the first derivative, $d_{t} = ( y_{t} - y_{t-1} )/T$ and the second derivative $s_{t} = ( d_{t} - d_{t-1} )/T$, respectively, where *T* denotes the time interval, that is, a week. For three influenza seasons in Fig. 2, $d_{t}$ abruptly fluctuates around zero before the onset of influenza outbreak. The second derivative $s_{t}$ also fluctuates around zero before the onset of outbreak. Then, we smooth the fluctuation of the first derivative through a seasonal cumulative mean given by

1 $$ \mu _{t} = \frac{1}{t} \sum _{t'=1}^{t} d_{t'}, $$

where $t' = 1$ denotes the first week of influenza season. As shown in Fig. 2, the difference between $d_{t}$ and $\mu _{t}$ increases at the onset of influenza outbreak. The above results are not confirmed only for three influenza seasons in Fig. 2, but for all seven influenza seasons (2009–2017) we extracted from the NHIS claims database. Therefore, similar to the KCDC’s warning criteria for the onset of influenza outbreak [20], we define the outbreak start week *t* as the condition

2 $$ d_{t} > \mu _{t} + k \cdot \sigma _{t} $$

is satisfied. Here, $\sigma _{t}$ is a seasonal cumulative standard deviation given by

3 $$ \sigma _{t} = \sqrt{\frac{1}{t-1} \Biggl\{ \sum_{t'=1}^{t} (d_{t'} )^{2} - \Biggl( \sum_{t' = 1}^{t} d_{t'} \Biggr)^{2} \Biggr\} }. $$

In Fig. 2(d)–(f), the blue dot shows $\mu _{t} + k \cdot \sigma _{t}$, and the outbreak start week *t* where the condition (2) is satisfied is marked by the blue circle in Fig. 2(a)–(c). In this study, the parameter value *k* is obtained through validation, which will be discussed later in this section. The KCDC defines the period from the 36th week of each year to the 35th week of the following year as an influenza season. Usually, the 36th week is around the end of August. Then, to sufficiently smooth the fluctuation of $d_{t}$, we set the week that includes July 1st as the first week of an influenza season, not the 36th week.

The outbreak starts week of *i*th city-county-district and *j*th age-group for each influenza season is computed as follows. If the condition ${d_{t}}^{i,j} > {\mu _{t}}^{i,j} + k \cdot {\sigma _{t}}^{i,j}$ is satisfied, then the *i*th city-county-district and *j*th age-group shows the start of influenza outbreak in week *t*. Here, ${d_{t}}^{i,j} = ({y_{t}}^{i,j} - {y_{t-1}}^{i,j} )/T$ where ${y_{t}}^{i,j}$ is the number of new patients in *i*th region and *j*th age-group. ${\mu _{t}}^{i,j}$ and ${\sigma _{t}}^{i,j}$ is the seasonal cumulative mean and standard deviation of ${d_{t}}^{i,j}$, respectively.

For validation of the TD, we apply it to ILI data [21] for finding the start of the influenza outbreak of the previous seasons. Also, the CUSUM and the EWMA control charts are applied for comparison. In the CUSUM, we compute the cumulative sum

4 $$ C_{t} = \max \bigl\{ 0, y_{t} - ( \mu _{0} + K ) + C_{t-1} \bigr\} , $$

where $y_{t}$ is the number of new patients in week *t* and $C_{0} = 0$. Here, $\mu _{0}$ is a target value, i.e., the mean of ILI for non-epidemic periods and $K = \delta \sigma / 2$ is the allowance where *σ* is a standard deviation of ILI for non-epidemic periods and *δ* is an amount of shift that we wish to detect in the unit of *σ*. Then, there is an onset of outbreak if $C_{t}$ exceeds the control limit *hσ*. For the CUSUM, we have two adjustable parameters *δ* and *h*. In the EWMA, the exponentially weighted moving average is defined as

5 $$ z_{t} = \lambda y_{t} + ( 1 - \lambda ) z_{t-1}, $$

where *λ* $( 0 < \lambda \le 1 )$ is a weighting factor. If $z_{t}$ is larger than the control limit, i.e., $\mu _{0} + L \sigma \sqrt{ \lambda / ( 2-\lambda ) \{ 1-(1-\lambda )^{2t} \} }$, then there exists an onset of influenza outbreak. As the same as the CUSUM, $\mu _{0}$ and *σ* are the mean and standard deviation of ILI for non-epidemic periods, respectively. For the EWMA, we have two adjustable parameters *λ* and *L*.

**Figure 3:**
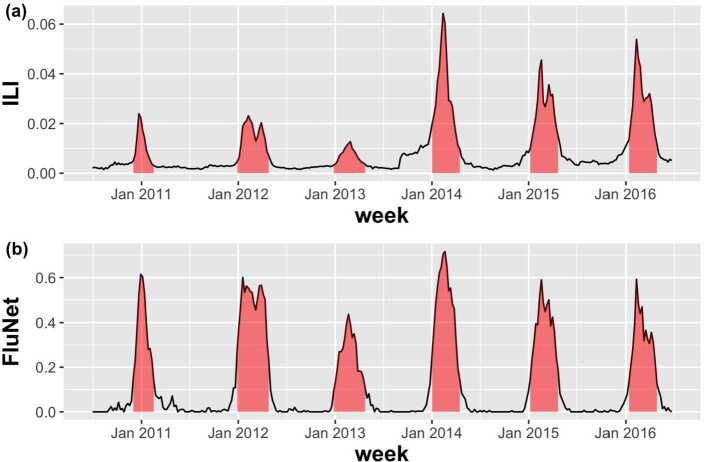
The proportions of ILI and positive influenza isolations. Fig. 3(**a**) shows the proportion of ILI patients among outpatients. The proportion of positive influenza isolations among specimens submitted to the reference laboratories is represented in Fig. 3(**b**)

**Figure 4:**
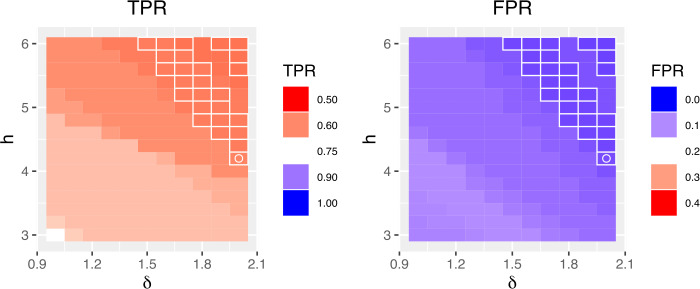
The TPR and FPR for the CUSUM. We show the TPR and FPR as a function of *δ* and *h*. Here, white boxes represent the parameter values where FPR ≤0.05, and the white circle indicates the maximum of TPR among FPR ≤0.05

**Figure 5:**
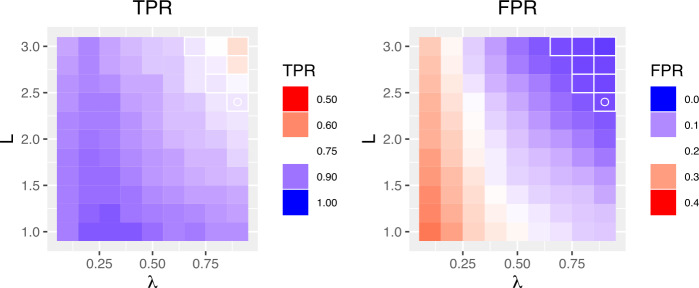
The TPR and FPR for the EWMA. We show the TPR and FPR as a function of *λ* and *L*. Here, white boxes represent the parameter values where FPR ≤0.05, and the white circle indicates the maximum of TPR among FPR ≤0.05

**Figure 6:**
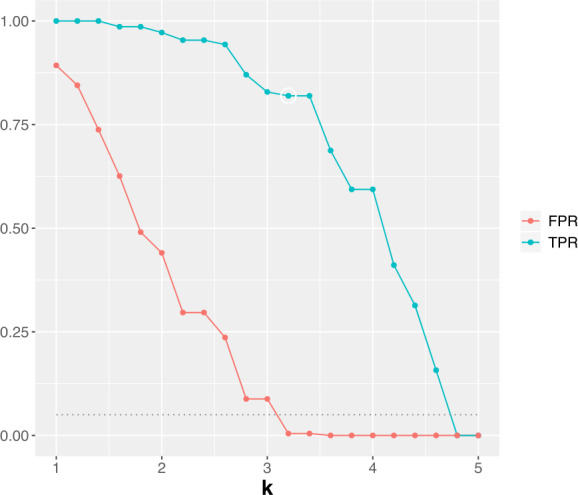
The TPR and FPR for the TD. We show the TPR and FPR as a function of *k*. Here, the white circle also represents the maximum of TPR among FPR ≤0.05

In this study, we set the epidemic periods for the previous influenza seasons according to the results of [12]. For validation of outbreak detection methods, the authors of [12] defined the start of influenza outbreak when the proportion of positive influenza isolations among respiratory specimens is larger than 20 percent of the maximum seasonal level. Here, we use the FluNet database [22] for respiratory specimens from 2010 to 2016 influenza seasons in South Korea. Figure 3 shows the proportions of ILI and positive influenza isolations. The red zone in Fig. 3 represents the epidemic periods for each season. Then, as varying adjustable parameters, we calculate the outbreak start week for each season and compute the true positive rate (TPR) and the false positive rate (FPR) for measuring the performance of CUSUM, EWMA, and TD. The TPR is defined as $n/N$ where *N* is the total number of weeks in the epidemic periods, and *n* is the number of weeks that we found among them. The FPR is described as $n'/N'$ where $N'$ is the total number of weeks outside the epidemic periods, and $n'$ is the number of weeks regarded as epidemic periods among them. For the CUSUM, Fig. 4 shows the results of level plots for TPR and FPR. In Fig. 4, white boxes represent the parameter values where FPR ≤0.05, and the white circle indicates the maximum of TPR among FPR ≤0.05. Here, we choose the best performance of detecting the onset of influenza outbreak as the parameter value where TPR shows the maximum and FPR ≤0.05. The results for EWMA are given in Fig. 5. Same as Fig. 4, white boxes represent the parameter values where FPR ≤0.05 and the white circle indicates the maximum of TPR among FPR ≤0.05. For the TD, Fig. 6 shows the results of TPR and FPR as a function of the parameter *k*. Note that there is only one adjustable parameter *k* for the TD. Here, the white circle also indicates the maximum of TPR among FPR ≤0.05. Table 1 summarizes the results of the maximum TPR among FPR ≤0.05. It shows that TD is more accurate than the CUSUM and the EWMA for early outbreak detection of influenza.

**Table 1:** The results of the maximum TPR among FPR ≤0.05. The TD is more accurate than the CUSUM and the EWMA for early outbreak detection of influenza

	FPR	TPR
TD	0.004	0.819
CUSUM	0.046	0.596
EWMA	0.046	0.778

## Results

**Figure 7:**
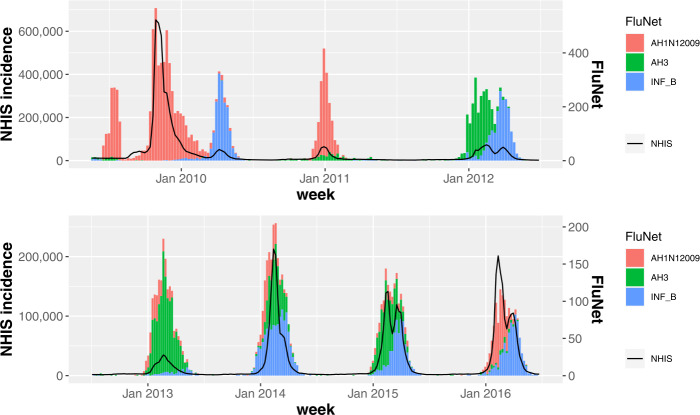
The time series of influenza incidence in South Korea (2009–2016). The black solid line shows the number of new patients within a week, which are extracted from the NHIS claims database. The histogram represents the numbers of influenza AH1N12009, AH3, and B virus isolates

First, let us investigate the incidence time series of South Korea during seven influenza seasons (2009–2017). In Fig. 7 the black solid line shows the number of new patients within a week, which are extracted from the NHIS claims database. Note that the axis ranges of Fig. 7(a) and (b) are different to improve the readability. We can see that every season, the onset and the peak of the outbreak occur at different weeks, and the height of the peak varies. There were the largest number of patients in the 2009–10 season and the incidence time series clearly shows a bimodal peak except for three influenza seasons (2010–11, 2012–13, 2013–14). Since the NHIS claims database does not include information on the type of influenza virus, we use the FluNet database to investigate which influenza viruses were isolated in South Korea. In Fig. 7, the histogram shows the numbers of influenza AH1N12009, AH3, and B virus isolates. We can see that the influenza A and B virus were detected in all seasons and the influenza A and B virus were predominant for the first and second peak, respectively, for the cases of the bimodal peak.

**Figure 8:**
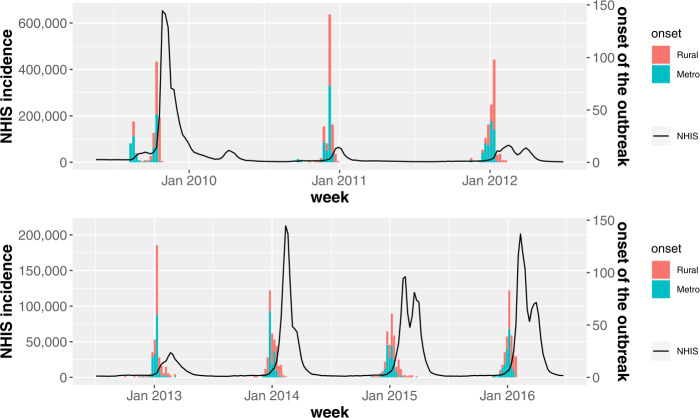
The histogram of the outbreak start weeks. The histogram shows the number of city-county-districts where the influenza outbreak has started in that week. As the same as Fig. 6, the black solid line represents the number of new patients

**Figure 9:**
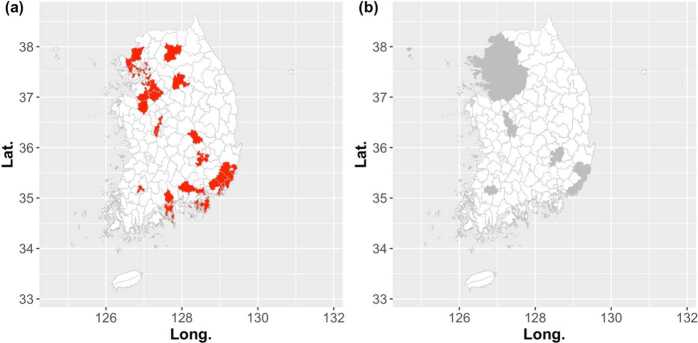
The regions that start earlier than other regions. The city-county-districts where the outbreak start week is earlier than or equals to $M_{s}$ for all seven influenza seasons are marked as red in Fig. 9(**a**). Here, $M_{s}$ is the median of outbreak start weeks. In Fig. 9(**b**), we show the metro and rural areas depicted in gray and white, respectively, for comparison

Then, we compute the outbreak start week of *i*th city-county-district for each influenza season by using the TD. Remind that the TD shows the best performance for detecting the onset of the influenza outbreak when the adjustable parameter *k* equals to 3.2 or 3.4. In this study, we set $k = 3.2$. Then, the histogram in Fig. 8 indicates the number of regions where the influenza outbreak has started in that week and the black solid line shows the number of new patients. Here, we divide the 250 city-county-districts into two groups. One is a metro area, which includes the capital region and each metropolitan city, and the other regions are bound to a rural area. To investigate which region the influenza outbreak starts earlier, we calculate the median of outbreak start weeks for each season, i.e., $M_{s}$. In Fig. 9(a), the city-county-districts where the outbreak start week is earlier than or equals to $M_{s}$ for all seven influenza seasons are marked in red. For comparison, in Fig. 9(b), we show the metro and rural areas depicted in gray and white, respectively. Although there are some rural regions among those marked in red, the results of Fig. 9 show that the metro area is earlier than the rural area for the start of the influenza outbreak.

**Figure 10:**
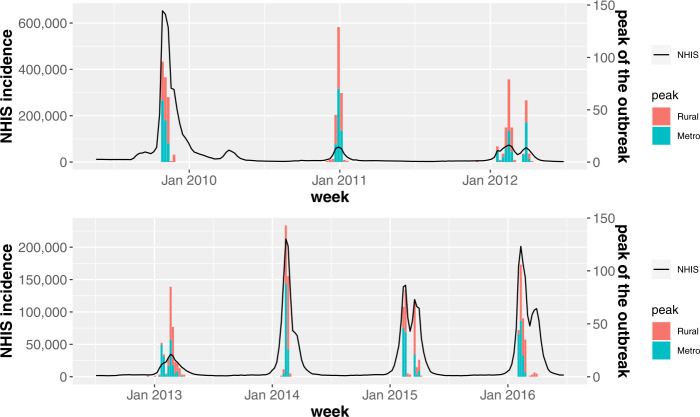
The histogram of the influenza peak weeks. The histogram represents the number of regions that have the influenza peak in that week. The black solid line represents the number of new patients

**Figure 11:**
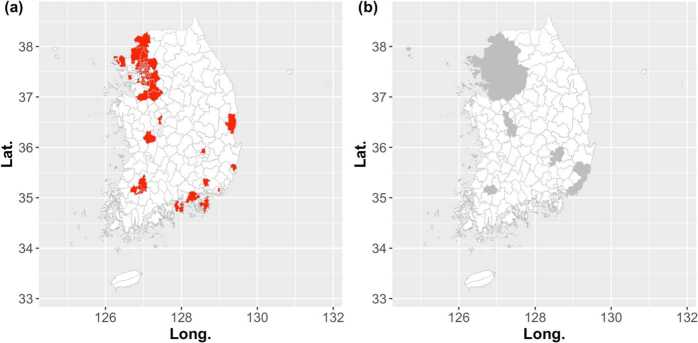
The regions that peak earlier than other regions. The city-county-districts where the influenza peak week is earlier than or equals to $M_{p}$ for all seven influenza seasons are marked as red in Fig. 11(**a**). Here, $M_{p}$ is the median of influenza peak weeks. In Fig. 11(**b**), we show the metro and rural areas depicted in gray and white, respectively, for comparison

For investigating which region the influenza peaks earlier, we compute the influenza peak week of *i*th city-county-district for each season. Note that the peak week is not calculated by the TD, and is obtained straightforwardly from the incidence time series in a retrospective manner. In Fig. 10 the histogram shows the number of regions that have the influenza peak in that week. As the same as Fig. 8, we divide the 250 city-county-districts into the metro and rural areas and the black solid line shows the number of new patients. Then, we compute the median of influenza peak weeks for each season, i.e., $M_{p}$. In Fig. 11(a), the city-county-districts where the influenza peak week is earlier than or equals to $M_{p}$ for all seven influenza seasons are marked in red. Similar to the start of the influenza outbreak, the results of Fig. 11 tell us that the metro area peaks earlier than the rural area.

**Figure 12:**
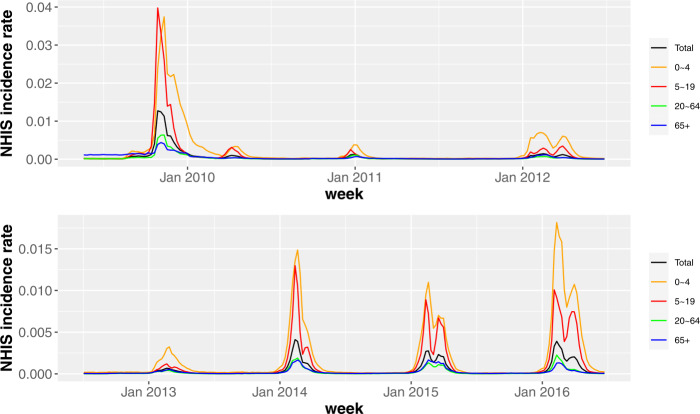
The incidence rate of influenza for each age-group in South Korea (2009–2016). We show the incidence rate of influenza, that is, the number of new patients within a week divided by the total number of population for that age-group

So far, we have investigated which region influenza starts and peaks earlier. Now, let us consider the incidence time series by age-group. Figure 12 shows the incidence rate of each age-group during seven influenza seasons (2009–2017). Note that, for improving the readability, we plot the incidence rate rather than the number of new patients by age-group. To investigate which age-group influenza starts earlier, we calculate the outbreak start week by the TD. The peak week for each group is straightforwardly counted from the incidence time series. The results are given in Tables 2 and 3. Except for the 2011–12 season when the height of the second peak of 5–19 age-group in the bimodal peak was higher than that of the first peak, the 5–19 age-group starts and peaks earlier than the other age-group.

**Table 2:** The influenza start weeks for each age-group. Here, we record Wednesday of that week. Except for the 2011–12 season, the 5–19 age-group starts earlier than the other age-group

	2009–10	2010–11	2011–12	2012–13	2013–14	2014–15	2015–16
0–4	2009-08-12	2010–11-24	2011-12-14	2013-01-09	2013-12-18	2014-12-24	2015-12-16
5–19	2009-08-12	2010-11-24	2011-12-21	2013-01-09	2013-12-11	2014-12-10	2015-12-16
20–64	2009-08-12	2010-12-08	2011-12-28	2013-01-09	2013-12-25	2014-12-24	2015-12-23
65+	2009-09-30	2010-12-15	2011-12-28	2013-01-09	2013-12-25	2014-12-24	2015-12-23

**Table 3:** The influenza peak weeks for each age-group. Here, we record Wednesday of that week. Except for the 2011–12 season when the height of the second peak of 5–19 age-group in the bimodal peak was higher than that of the first peak, the 5–19 age-group peaks earlier than the other age-group

	2009–10	2010–11	2011–12	2012–13	2013–14	2014–15	2015–16
0–4	2009-11-11	2010-12-29	2012-02-08	2013-02-27	2014-02-19	2015-02-18	2016-02-10
5–19	2009-10-28	2010-12-22	2012-03-28	2013-02-20	2014-02-12	2015-02-11	2016-02-03
20–64	2009-11-11	2010-12-29	2012-02-15	2013-02-20	2014-02-19	2015-02-18	2016-02-10
65+	2009-11-04	2011-01-05	2012-02-15	2013-02-27	2014-02-19	2015-02-18	2016-02-17

## Discussions

For control policies to an influenza outbreak, it can be helpful to understand the characteristics of regional and age-group-specific spread. However, in South Korea, there has been no official statistic related to it. Therefore, in this study, we have extracted the time series of influenza incidence, i.e., the number of new patients by region (250 city-county-districts) and age-group (0–4, 5–19, 20–64, 65+) within a week from the NHIS claims database, which consists of all medical and prescription drug-claim records for all South Korean population. The number of cases of influenza (2009–2017) is 12,282,356. Note that previous studies on the spatiotemporal spreading pattern of influenza used sentinel surveillance data rather than the whole incidence data, and only considered the propagation of epidemic peak rather than the onset of an outbreak. Unlike these previous studies, we have used the whole incidence data for all South Korean population and investigated the propagation of the onset of an influenza outbreak as well as the peak. For computing the start of an influenza outbreak by region and age-group, we have proposed the TD. The TD does not require information on the previous non-epidemics periods and detect the onset of an outbreak as a sudden change in the time derivative of incidence. The results on TPR and FPR confirm that the TD is more accurate than the CUSUM and the EWMA for early outbreak detection of influenza. Then, we have shown that the metro area and 5–19 age-group are earlier than the rural area and other age-groups for the start of the influenza outbreak. Also, the metro area and 5–19 age-group peak earlier than the rural area and other age-groups. As of July 2020, during the COVID-19 pandemic, it seems appropriate to mention the following. The results of [8] and our study shows that children start and peak earlier than other age-groups for the outbreak of influenza. However, as of July 2020, COVID-19 seems to be uncommon in children [23, 24]. Since the COVID-19 pandemic is still in progress, the results may change in the future, but influenza and COVID-19 have shown different aspects of the prevalence of children.

So far, the early warning for influenza outbreak by region and age-group has not been available in national influenza surveillance systems of South Korea. But, due to the TD, the early warning by region and age-group can be possible. Also, when we need to operate influenza surveillance systems with limited resources, we can focus on specific regions and age-group, which is earlier than others for the start of influenza outbreak. The NHIS claims database we used requires at least six months of processing time to utilize them after the occurrence of medical and prescription drug-claim records. Therefore, it is not possible to operate a real-time early warning system for influenza outbreak using the NHIS claims database. However, by applying the TD for real-time data, such as the drug utilization review of Health Insurance Review and Assessment service of South Korea or National Emergency Department Information System of South Korea, real-time early warnings can be possible. Then, our results would be helpful to design a surveillance system for timely early warning of an influenza outbreak in South Korea.
